# A Multi-Objective Optimization of Neural Networks for Predicting the Physical Properties of Textile Polymer Composite Materials

**DOI:** 10.3390/polym16121752

**Published:** 2024-06-20

**Authors:** Ivan Malashin, Vadim Tynchenko, Andrei Gantimurov, Vladimir Nelyub, Aleksei Borodulin

**Affiliations:** 1 Artificial Intelligence Technology Scientific and Education Center, Bauman Moscow State Technical University, 105005 Moscow, Russiavladimir.nelub@emtc.ru (V.N.);; 2 Scientific Department, Far Eastern Federal University, 690922 Vladivostok, Russia

**Keywords:** multi-objective optimization, neural networks, textile polymer composites, physical properties’ prediction

## Abstract

This paper explores the application of multi-objective optimization techniques, including MOPSO, NSGA II, and SPEA2, to optimize the hyperparameters of artificial neural networks (ANNs) and support vector machines (SVMs) for predicting the physical properties of textile polymer composite materials (TPCMs). The optimization process utilizes data on the physical characteristics of the constituent fibers and fabrics used to manufacture these composites. By employing optimization algorithms, we aim to enhance the predictive accuracy of the ANN and SVM models, thereby facilitating the design and development of high-performance textile polymer composites. The effectiveness of the proposed approach is demonstrated through comparative analyses and validation experiments, highlighting its potential for optimizing complex material systems.

## 1. Introduction

Polymer composite materials (PCMs) are composite materials consisting of two or more components, where the final mixture exhibits properties superior to those of each individual constituent material [1]. PCMs are indispensable in contemporary engineering and industrial applications owing to their exceptional characteristics, including but not limited to lightweight design [2], high strength [3], and resistance to corrosion [4,5]. However, the development of effective polymer composites necessitates the consideration of diverse factors [6], notably encompassing composition and structure.

Attaining the desired attributes of polymers mandates the application of multifaceted approaches, including choosing optimal raw components [7], with the necessary chemical and physical properties being a primary consideration. This may involve selecting different polymers [8], fillers [9], reinforcing materials [10], and additives [11]. Careful tuning of the composition and proportions [12] of components in the material can significantly impact its properties. This could involve altering the concentration of the polymer components [13,14]. Additionally, the chemical modification of the polymers [15,16] can be used to change their properties. This may involve introducing functional groups [17] and changing the molecular weight [18] and crystalline structure [19] of the polymer. Various treatments such as extrusion [20], injection molding [21], compression molding [22], and filament winding [23] can be used to shape and enhance the properties of polymer materials. Furthermore, the addition of nanoparticles [24] or nanotubes [25] to polymer composites can significantly improve their mechanical, thermal, and electrical properties [26].

The specific physical characteristics of polymers become imperative to meet precise functional requisites across various applications [27]. These characteristics may encompass mechanical properties such as tensile strength [28], flexibility [29], and impact resistance [30], alongside thermal attributes like heat resistance [31] and thermal conductivity [32]. Thus, a comprehensive understanding of the polymer composite composition and structure is pivotal, facilitating the customization of the properties to align with distinct application demands.

One of the modern approaches to achieving the desired properties of PCMs involves the application of machine learning (ML) techniques. ML algorithms, such as neural networks [33], support vector machines [34], and random forests [35], can analyze complex datasets comprising the material composition, processing parameters, and desired properties to identify intricate relationships and patterns. By leveraging these relationships, ML models can predict the properties of PCMs [36], optimize material formulations, and expedite the development process. This approach has been actively discussed and extensively researched in the scientific literature.

Fontes et al. [37] showcased the effectiveness of Deep Neural Networks (DNNs) in crafting a data-driven failure model for Fiber-reinforced Polymer (FRP) composite materials. Using experimental failure data from the literature, a fully connected DNN with 20 input units and one output unit was trained. The inputs included the laminate layup sequence, the lamina properties, and the loading conditions, while the output was the failure vector length. Comparative analysis with conventional theories such as Tsai–Wu [38], Cuntze [39], and Pinho theory [40] indicated the superior performance of the DNN in fitting the experimental data. Its ability to handle higher order polynomials makes it a valuable tool for predicting FRP composite laminate failure.

Fahem et al. [41] investigated the impact of porosity on the mechanical properties of Glass Fiber-Reinforced Polymer (GFRP) [42,43] through experimental and numerical analyses. The material characterization included a three-point bending test, while the finite-element modeling explored various air bubble scenarios. The results revealed a significant reduction in load as the bubble size increased. Additionally, an artificial neural network-Enhanced Jaya Algorithm (ANN-E JAYA) [44] predicted the tensile load reduction based on the crack lengths from an Extended finite-element method (XFEM) [45]. A comparison with other algorithms, including the Jaya Algorithm (JAYA) [46] and particle swarm optimization (PSO) [47], demonstrated the superior accuracy of ANN-E JAYA.

Nguen et al. [48] explored the impact of the cure-induced size effect on stress development and tensile transverse failure response in thermoset composite materials. Thick structures, combined with low polymer conductivity, may result in spatially varying temperature fields, affecting the property distribution and residual stress. The study employed a thermo-chemo-mechanical finite-element framework integrated with a crack band model. The cure model considered the kinetics and heat generation, while a neural network-based constitutive model captured the matrix mechanical property evolution.

Gupta et al. [49] proposed an ML model to precisely quantify the mechanical properties of FRP composites for optimal structural design. Using microstructural images as the input, the model visualizes the stress components, specifically $S_{11}$, with high accuracy. The training data were obtained from the FEM analysis of short carbon fiber-filled specimens using a Representative Area Element (RAE) approach [50]. The study demonstrated the robustness of a pix2pix [51] deep learning Convolutional Neural Network (CNN) model in predicting the stress fields. By focusing on the chronological development of the CNN model, the paper outlined a promising approach to efficiently predict full-field stress maps in fiber-reinforced composite specimens, reducing the time and cost associated with traditional methods.

El [52] used a Deep Recurrent CNN (DCRN) [53] to predict the full non-linear response of composite materials. The framework relies on a Representative Volume Element [54] (RVE) database, encompassing the composite layups, defects, and loading conditions. It incorporates various sources of material non-linearity, including matrix damage, delamination, fiber failure, and shear non-linearity. The proposed DCRN architecture combines convolutional layers for spatial feature detection with Long Short-Term Memory layers for the material loading history dependencies.

Zhang et al. [55] focused on enhancing the thermal protection performance of PCMs for re-entry vehicles. A thermal response model without surface recession was developed to simulate the ablation process. Using ML, the relationship between the piecewise porosity distribution and bondline temperature was explored based on simulated data. Optimal porosity distributions were obtained, leading to a reduction in the bondline temperature by 17.61 K, highlighting the potential of rational porosity optimization to improve material utilization rates.

Song et al. [56] presented an approach using digital material twins to analyze the mechanical performance of woven composites, particularly damage and failure behaviors. Addressing challenges in segmenting low-contrast digital images and reconstructing 3D braided structures, a ResL-U-Net CNN was proposed. The network incorporates the leaky-ReLU [57] activation function for efficiency and employs residual structures to enhance robustness and accuracy. The results demonstrated that the simulations accurately depicted the mechanical performance of GFRP, including the damage locations and material failure patterns.

Li et al. [58] introduced a DL fusion model for predicting the material properties of carbon fiber monofilaments [59] by simultaneously analyzing textual and visual data. Utilizing the greedy-based generation [60] (GBG) algorithm, 1200 stochastic microstructures were generated, and the statistical representations were determined using two-point statistics. The macroscopic properties were calculated via micro-scale finite-element simulation. The developed hybrid CNN-MLP fusion model achieved impressive average testing $R^{2}$ values for various mechanical properties of carbon fibers.

Doddashamachar et al. [61] predicted the dielectric properties of polypropylene composites reinforced with banana fiber using an ANN. Composites were prepared according to ASTM standards with varying banana fiber volume fractions [62]. The dielectric characteristics were determined using an impedance analyzer. The ANN, trained with the ReLU activation function, showed accurate prediction of the dielectric properties.

Amor at al. [63] provided an overview of computational intelligence (CI) modeling methods for lightweight composite materials (LWCMs). CI facilitates material data science tasks such as imaging, feature identification, prediction, and design optimization, enhancing LWCM quality through constituent optimization.

Mukhopadhyay [64] explored the use of an ANN in predicting the mechanical properties and behaviors of textile composite materials, including the static and dynamic mechanical properties, time-dependent properties like creep and stress relaxation, fatigue prediction, wear simulation, and crack detection. The discussion highlighted recent developments and applications of ANNs in the field of fiber-reinforced composites, emphasizing the importance of accurately modeling composite properties for engineering applications.

This article aims to bridge the existing gap in knowledge by predicting the physical characteristics of textile polymer composite materials (TPCMs) [65] based on a dataset encompassing the mechanical properties of fabrics and yarns in both the warp and weft directions, which constitute these fabrics. Through this analysis, the study delves into optimization methods aimed at fine-tuning the hyperparameters and selecting appropriate architectures for machine learning (ML) models. The primary focus is on exploring the effectiveness of optimization methods such as multi-objective particle swarm optimization [66] (MOPSO), Non-dominated Sorting Genetic Algorithm II [67] (NSGA-II) [67], and Strength Pareto Evolutionary Algorithm 2 [68] (SPEA2) in optimizing ML models like support vector machines [69] (SVMs) and ANNs to maximize accuracy and minimize inference time.

## 2. Materials and Methods

### 2.1. Dataset Description

To evaluate the tensile, compressive, and other properties of TPCMs, we prepared specially designed specimens in the form of strips measuring 10 × 20 cm. These specimens were securely mounted in a universal testing machine, the QUASAR 50 universal testing machine (Galdabini, Cardano al Campo, Italy), which applies mechanical forces to the material.

During a tensile test, the specimen was subjected to gradually increasing axial force until it ruptured, allowing us to assess its tensile strength and elongation properties. Conversely, in a compressive test, the specimen was compressed along its length by applying opposing forces at its ends, enabling the characterization of its compressive strength and modulus.

**Figure 1:**
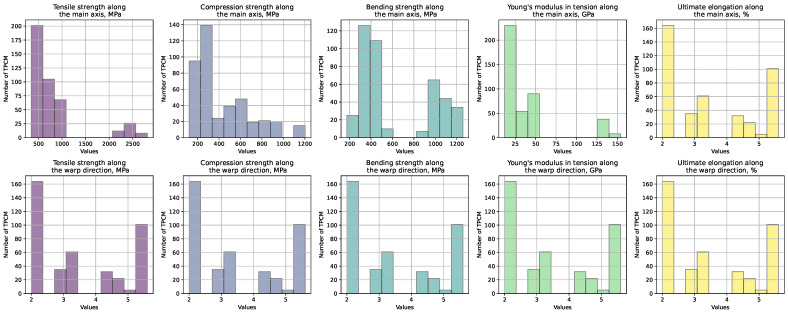
Physical properties of textile PCMs such as the tensile, compressive, and bending strengths and the modulus of elasticity in tension along the warp and weft directions, respectively, for the considered specimens.

Figure 1 illustrates histograms depicting the distribution of the physical characteristics of textile PCMs to showcase key physical properties such as the tensile, compressive, and bending strengths, the percentage of elongation, and the modulus of elasticity in tension along the warp and weft directions, respectively, for each mentioned characteristic.

**Figure 2:**
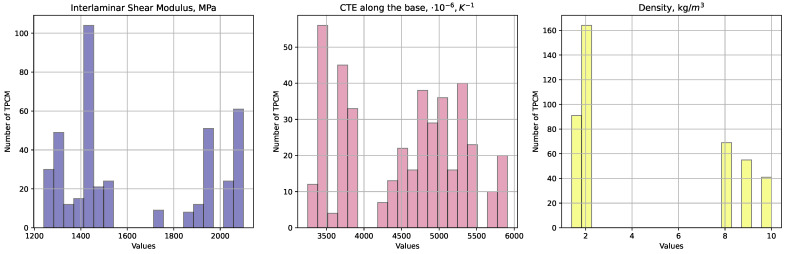
Distribution of the interlaminar shear modulus, coefficient of linear thermal expansion (CTE) along the warp direction, and density for the considered specimens.

Figure 2 showcases histograms representing the spread of physical attributes like the interlaminar shear modulus, coefficient of linear thermal expansion along the warp direction, and density.

The study involved conducting a total of 420 measurements on samples to investigate 11 mentioned physical characteristics across various types of TPCMs and their corresponding constituents. Specifically, the materials under investigation included the following:

1. Basalt plastic, designated as TBK-100 [70,71], is a composite material comprising basalt fibers [72] as the reinforcement phase embedded in a polymer matrix. Basalt fibers are derived from natural volcanic rock [73] and have high tensile strength and resistance to temperature variations. TBK-100 finds application in construction [74]. The weave pattern of these samples is canvas.
2. Fiberglass-reinforced plastic, commonly known as fiberglass [75], is a composite material composed of fine glass fibers embedded in a polymer matrix, typically epoxy or polyester resin [76]. This material exhibits a high strength-to-weight ratio [77], excellent corrosion resistance [78,79], and dimensional stability [80], making it suitable for applications requiring durability and structural integrity [81]. We considered types such as T-10 [82], T-13 [83], T-11 [84], T-SU 8/3(VMP)-78 [85], and T-25 [86]. The fabric construction of these samples predominantly consisted of canvas [87] and satin weaves [88].
3. Carbon fiber-reinforced plastic, also known as carbon fiber composite or carbon composite [89], consists of carbon fibers infused in a polymer matrix, often epoxy resin. This material offers exceptional strength, stiffness, and lightweight properties, making it ideal for aerospace [90], automotive [91], and sporting goods [92] applications where weight reduction and high performance are critical. We considered CC245 [93], CC206 [94], T700SC [95], UMT49 [96], UT-900-3 [97], HTS45 [98], and IMS65 [99]. The weave pattern observed in these samples primarily included twill [100] and unidirectional [101] weaves.
4. Aramid fiber-reinforced plastic, or aramid composite [102], incorporates aramid fibers, such as Kevlar^®^ [103], as the reinforcing component in a polymer matrix. Aramid fibers are known for their exceptional strength, stiffness, and resistance to impact and abrasion [104]. Aramid composites offer high tensile strength, heat resistance, and low weight, making them suitable for ballistic protection [105]. We considered varieties like T-43-76 [106], Satin 5/3 [107], Satin 8/3 [108], T-42-78 [109], and T-42/1-76 [110]. For this type of TPCM, the weave patterns also included canvas and satin.

**Figure 3:**
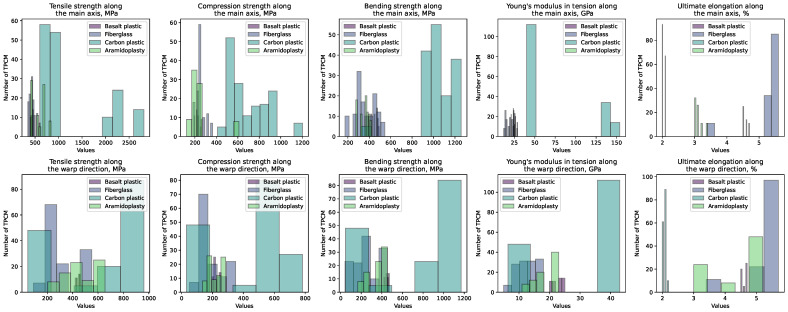
Histograms depicting the distribution of the physical characteristics for TPCMs grouped by type: basalt plastic, fiberglass, carbon plastic, and aramid plastic. Each subplot illustrates the distribution of the following characteristics along the main and warp directions: tensile strength, compression strength, bending strength, Young’s modulus, and ultimate elongation.

To analyze the distribution of the key physical characteristics across various types of TPCMs, we utilized histograms. These materials were categorized into basalt plastic, fiberglass, carbon plastic, and aramid plastic. Each histogram in Figure 3 showcases the distribution of specific characteristics, such as the tensile strength [111], compression strength [112], bending strength [113], Young’s modulus [114], and ultimate elongation [115], along both the main axis and the warp direction.

**Figure 4:**
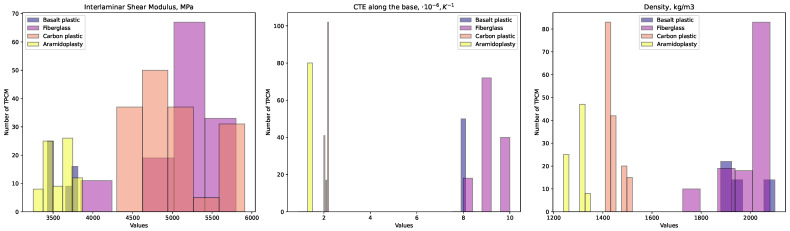
Histograms of physical attributes for TPCMs grouped by type: basalt plastic, fiberglass, carbon plastic, and aramid plastic for the distributions of the interlaminar shear modulus, CTE, and density.

Figure 4 illustrates the variations in the distribution of the interlaminar shear modulus [116], CTE [117], and density [118].

Unlike Figure 1 and Figure 2, Figure 3 and Figure 4 feature histograms with variable bar widths. This variation in bar thickness aims to highlight the distribution density of different data points. Thicker bars denote a higher number of TPCMs or the concentration of data within a range, while thinner bars represent a lower number of TPCMs. This method offers a more nuanced understanding of the data distribution compared to uniform bar widths.

**Figure 5:**
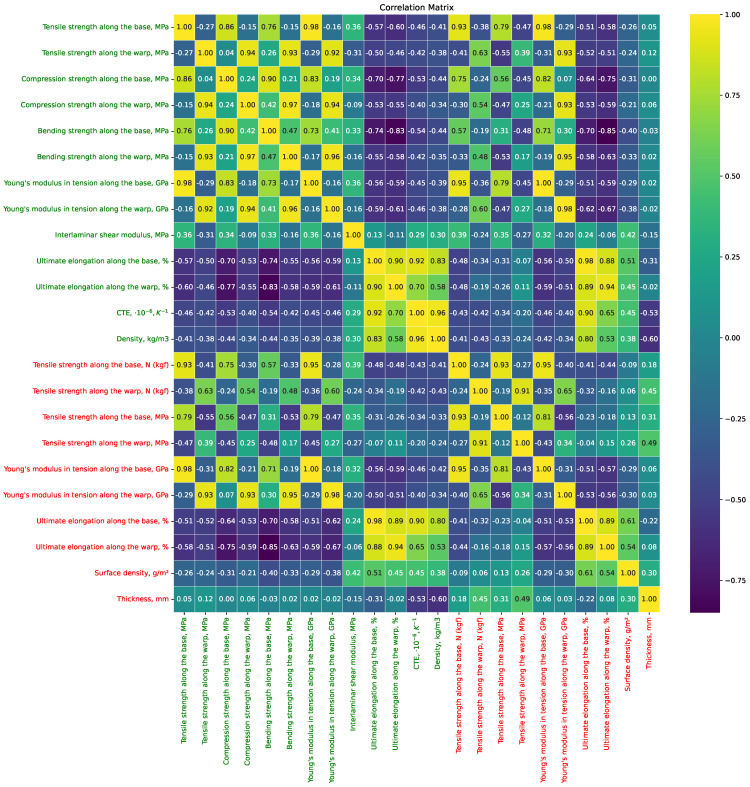
Correlation matrix of physical properties of TPMCs (highlighted in green) and fabric properties (highlighted in red), from which the samples are derived.

**Figure 6:**
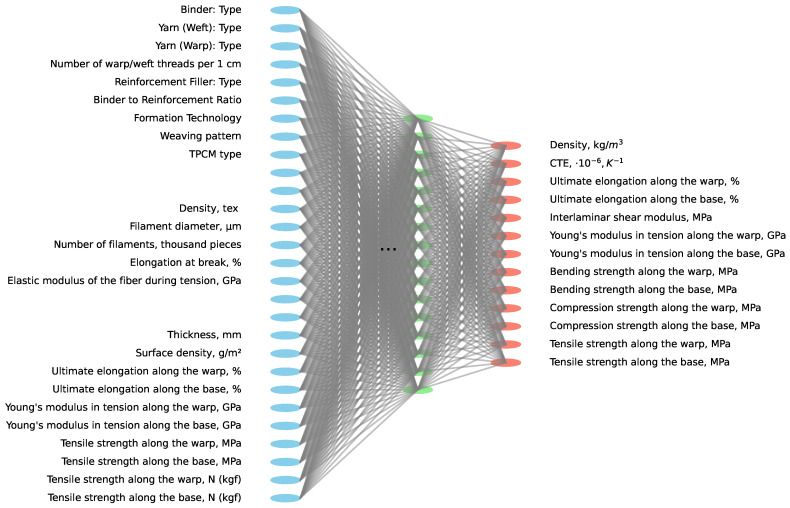
Illustration of a hypothetical neural network architecture designed to predict the physical characteristics of TPCMs: blue dots as inputs, red dots as outputs.

Additionally, an analysis of the correlation matrix was conducted, reflecting the relationship between the physical characteristics of the TPCMs and the properties of the fabrics from which these samples are made (Figure 5). These relationships could be the basis for developing predictive models that estimate the behavior of composite materials based on the fabric properties and can aid in quality control during the manufacturing process. Figure 5 highlights the numerical input features of the dataset in green and the output features in red, as also depicted in Figure 6. From this correlation matrix, one can observe patterns such as the correlation between the parameters related to the yarns, such as the tensile strength along the base and warp, compression strength, bending strength, and Young’s modulus. These correlations exhibit a chessboard-like pattern, indicating that the values along the base correlate more strongly with each other than with the values along the warp. This pattern is similarly observed for the output parameters, such as those pertaining to finished TPCM products, with the same names, but for finished products.

To build a model for predicting the physical characteristics of polymer composite materials, we utilized neural networks, which allow modeling complex non-linear relationships between the material components and their properties. Neural networks consist of multiple layers of neurons that process input data and provide predictions.

To optimize the performance of neural networks, we applied hyperparameter optimization methods such as Grid Search, Random Search, and Bayesian Optimization. These methods enabled us to automatically find optimal values for the model hyperparameters such as the number of hidden layers, the number of neurons in each layer, the learning rate, and others.

### 2.2. Model Development

To predict the physical characteristics of the TPCMs, an SVM and ANN were selected as the optimization methods. The SVM is capable of handling both linearly and non-linearly separable data and is resistant to overfitting. Due to the small number of hyperparameters, this simplifies the optimization process and parameter tuning. The ANN possesses the ability to adapt to various types of data and tasks, achieving high accuracy when properly configured.

To further address the task at hand, it is necessary to determine the selected optimization methods. NSGA-2 is used for multi-objective optimization problems, aiming to find a set of optimal solutions known as the Pareto set [119,120]. NSGA-2 is designed for problems with multiple optimization criteria, ensuring diversity in the population to prevent premature convergence and achieve uniform coverage of the Pareto set.

SPEA-2 is an evolutionary algorithm also utilized for multi-criteria optimization. It ranks solutions based on their strength and distance from other solutions to effectively select the best solutions for constructing the Pareto set [121,122].

MOPSO is a variant of the particle swarm optimization (PSO) method, also applied to multi-criteria optimization. It iteratively updates the position and velocity of particles in the parameter space [123] based on the best solutions and information exchange between particles.

Python was chosen for implementing the algorithm due to its powerful capabilities in machine learning and optimization. We utilized the scikit-learn library for SVM model handling and PyGMO for the MOPSO algorithm. Data preprocessing was conducted using pandas and numpy, followed by splitting into training and testing sets. The optimization space encompassed the linear [124], polynomial [125], or RBF [124] kernel types and a regularization parameter [126] for the SVM. For multi-objective optimization, we defined minimizing the inference time and maximizing the model accuracy as the objectives. The inference time is computed as the average inference time on the test dataset for each SVM model. Accuracy is computed as the accuracy on the test dataset for each SVM model. The MOPSO algorithm was implemented using PyGMO [127], involving initialization, updating, and evaluating the solutions. The solution updating followed the principles of dominance and best solution selection. The algorithm continued until reaching a specified number of iterations or stopping criteria, after which the best solution was selected based on multi-objective optimization.

Figure 6 shows the schematic representation of a potential neural network architecture for predicting the physical properties of TPCMs.

The input features include the properties of both the fabric and thread. The fabric properties encompass parameters such as the elastic modulus of the fiber during tension (GPa), elongation at break (%), number of filaments (thousand pieces), filament diameter (μm), and density (tex). Additionally, thread properties include the tensile strength along the base and warp (N and MPa), Young’s modulus in tension along the base and warp (GPa), ultimate elongation along the base and warp (%), surface density (g/m^2^), and thickness (mm). Categorical features such as the TPCM type (like T-43-76, Satin 5/3, Satin 8/3, etc.), weaving pattern, formation technology, binder-to-reinforcement ratio, reinforcement filler type, number of warp/weft threads per 1 cm, yarn type for warp and weft, and binder type were also included as inputs. The network predicts the parameters of the final TPCM product, including the tensile strength, compression strength, bending strength, Young’s modulus, interlaminar shear modulus, ultimate elongation, and CTE.

## 3. Results

**Figure 7:**
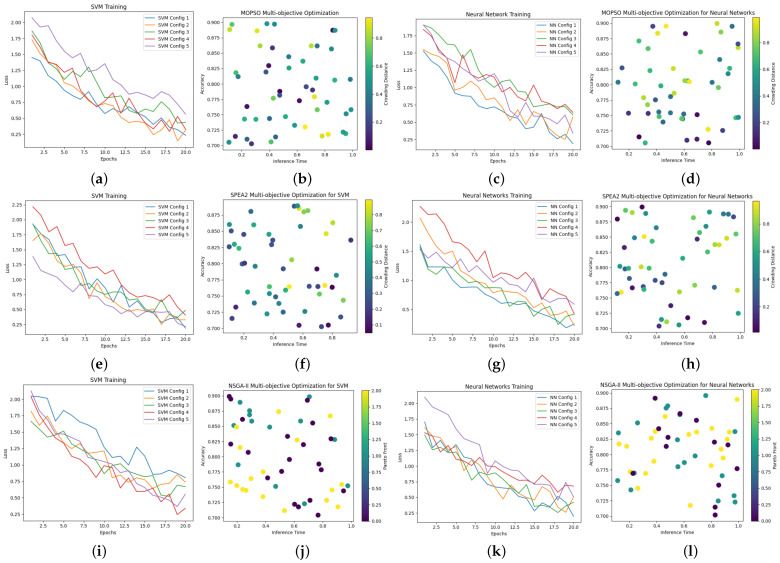
Evolution of loss curves and optimization parameter space for hyperparameter tuning in the ANN and SVM using the MOPSO (**a**–**d**), SPEA2 (**e**–**h**), and NGSA-II (**i**–**l**) optimization methods.

Our study investigates the efficacy of employing metaheuristic optimization algorithms, specifically MOPSO, SPEA2, and NSGA-II, for hyperparameter tuning in the ANN and SVM for predicting the physical properties of TPCM samples based on their fabricated components. In Figure 7, we present the dynamic evolution of the loss curves for the five best models for each optimization case alongside the exploration of the optimization parameter space.

This figure encapsulates the iterative optimization process, illustrating how these algorithms navigate the complex landscape of hyperparameters to achieve optimal performance in both the ANN and SVM models. Such analysis provides valuable insights into the comparative effectiveness of these optimization techniques, shedding light on their suitability for enhancing the predictive capabilities of machine learning models.

The ANN model architecture was constructed based on the genetic algorithm’s (GA) [128] individual representation for the optimizers NSGA-II and SPEA2. The architecture consisted of densely connected layers with leaky-ReLU [57] activation functions. The number of layers was dynamically determined, but capped at a predefined maximum. Each layer’s neuron count was constrained within specified bounds. The model was trained using k-fold cross-validation [129] to mitigate overfitting, and its performance was evaluated based on the root-mean-squared error (RMSE) [130] metric. Key parameters such as the population size [131] specified the number of individuals (ANN architectures) in each generation of the genetic algorithm. In our case, the population size was set to 50. The crossover probability [132] determined the likelihood of crossover occurring between two parent individuals during reproduction. We set a value of 0.7; there was a 70% chance of crossover. The mutation probability [133] represents the probability of mutation, which introduces small random changes to individual genomes. We set a value of 0.3, indicating a 30% chance of mutation. The number of generations [134] indicates how the GA will evolve the population. In our scenario, the algorithm ran for 30 generations. The number of training epochs [135] (iterations over the entire dataset) during the training of each ANN was 100 epochs. The minimum number of neurons allowed in a single layer of the ANN was set to 2 neuron, and the maximum was 32 neurons in increments of 2. We defined the minimum and maximum learning rates as 0.05 and 0.2, respectively, which control the step size during gradient descent optimization. The learning rate [136] typically falls within a predefined range to balance training stability and convergence speed. The maximum number of layers allowed in the ANN architecture was constrained to five.

For MOPSO, the fitness function evaluates a solution (particle position) using SVR and the ANN and calculates the RMSE for each target variable. The algorithm runs the MOPSO algorithm for a specified number of iterations as 50 with 20 particles each time, updating the particles’ positions and velocities based on their personal best and global best positions.

**Figure 8:**
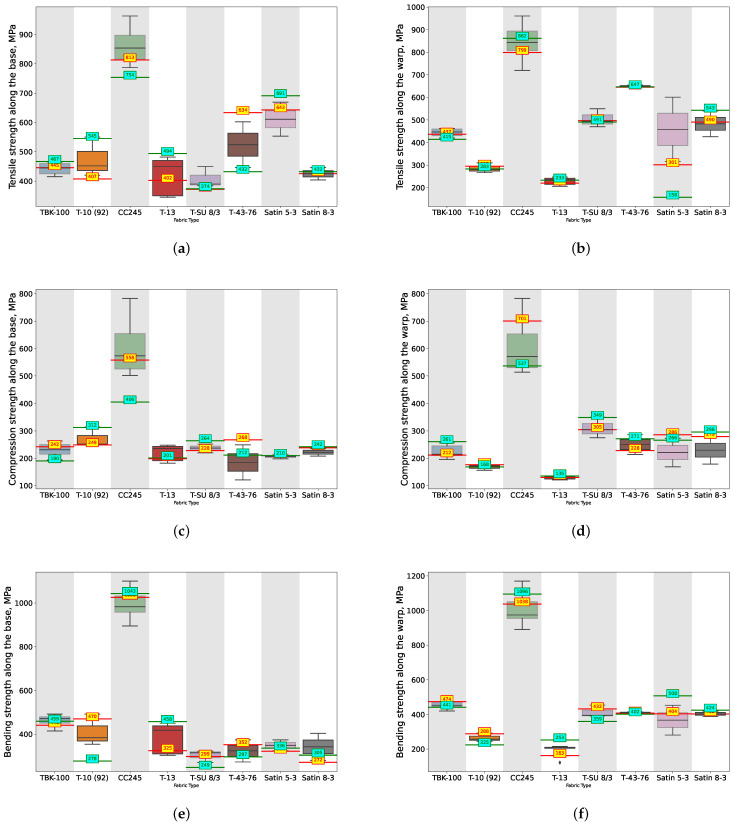
Whisker charts for selected types of TPCMs depending on physical property: (**a**) Tensile strength along the base, (**b**) Tensile strength along the warp, (**c**) Compression strength along the base, (**d**) Compression strength along the warp, (**e**) Bending strength along the base, (**f**) Bending strength along the warp with predictions made by the best architectures of the ANN (digits in yellow) and SVM (digits in cyan), architectures optimized using the MOPSO and NSGA-II algorithms, respectively.

**Figure 9:**
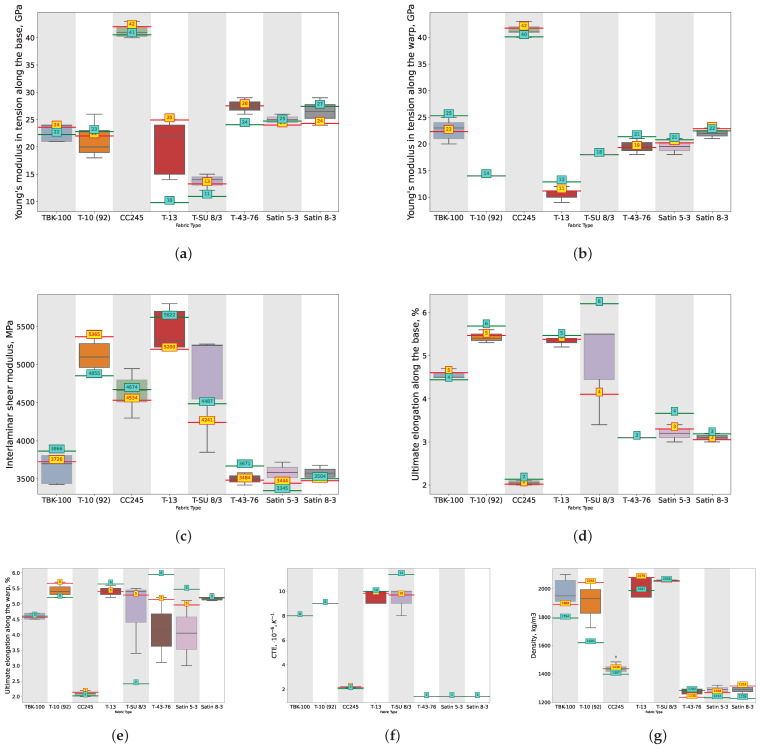
Whisker charts for selected types of TPCMs charts for selected types of TPCMs depending on physical property: (**a**) Tensile strength along the base depending on physical property: (**a**) Young’s modulus in tension along the base, (**b**) Young’s modulus in tension along the warp, (**c**) Interlaminar shear modulus, (**d**) Ultimate elongation along the base, (**e**) Ultimate elongation along the warp, (**f**) CTE, (**g**) Density with predictions made by the best architectures of the ANN (digits on yellow) and SVM (digits on cyan), architectures optimized using the MOPSO and NSGA-II algorithms, respectively.

The predictive performance of the ANN and SVM, optimized using heuristic algorithms, in predicting the physical characteristics of the TPCMs is shown in Figure 8 and Figure 9 for all 13 output features highlighted in red in Figure 6. These Figure 8 and Figure 9 present the median values as whisker plots for some of the polymer grades, while the yellow (for the ANN) and cyan (for the SVM) markers indicate the values proposed by the best ML models, as summarized in Table 1. For convenience, we also provide a table summarizing the optimization parameters for the ANN and SVM using MOPSO, SPEA2, and NSGA-II.

The findings presented in Figure 9 are a continuation of those shown in Figure 8. Together, they form a series of 13 subfigures, each representing a numerical physical characteristic of the TPCMs distributed across different TPCM grades (types), like TBK-100, T-10 (92), CC245, T-13, T-SU 8/3, T-43-76, Satin 5-3, and Satin 8-3.

The boxplot whisker chart provides a visual representation of the distribution of the values for each physical characteristic across the selected grades of TPCMs for the real dataset. Each boxplot illustrates the median, shown as a line within the colored area of the whisker, and the quartiles, represented by the boundaries of the colored area. Additionally, the chart depicts potential outliers and extreme values as horizontal lines outside the colored area. These elements are presented for each physical characteristic based on the TPCM grade, enabling a detailed comparison of the data distribution across different polymer grades.

Specifically, these grades are those for which experiments were conducted more than 10 times. On each of the whisker plots, the predicted values generated by modeling with the ANN optimized using MOPSO are indicated in yellow digits, and for the SVM optimized using NSGA II represented in cyan digits. These predictions are based on the architecture hyperparameters provided in Table 1. Such analysis offers insights into the efficacy of different machine learning approaches in capturing the complex relationships inherent in the TPCM properties.

## 4. Discussion

For predicting the physical characteristics of TPCMs, we employed multi-objective optimization algorithms to develop predictive models for the TPCM properties based on the constituents that comprise these materials.

Our choice was motivated by several factors, among which the prominent one was the robustness and extensive application of MOPSO, NSGA II, and SPEA2 in optimization tasks. These algorithms exhibit efficient search capabilities and adeptly manage multiple conflicting objectives [137] concurrently. Furthermore, their evolutionary nature renders them well-suited for navigating high-dimensional parameter spaces. Iteratively exploring these spaces, they refine solutions progressively, converging towards optimal or near-optimal solutions. Also, these algorithms provide a diverse set of solutions, allowing us to explore the trade-offs between different objectives and select the most suitable model configurations based on the specific requirements of the application.

To address classical ML issues such as avoiding local optima [138], heuristic algorithms offer several advantages. They use a population of potential solutions, allowing the exploration of multiple regions of the parameter space simultaneously and reducing the likelihood of becoming stuck in local optima [139]. Additionally, they balance different aspects of model performance, such as accuracy and generalization [140], leading to more reliable solutions. Initializing particles with diverse positions helps cover a broader area of the search space, increasing the chances of finding global optima.

To ensure the robustness of our dataset, we conducted a validation using information obtained from open sources. Several of these sources are listed in Section 2.1. By comparing and verifying the information against established sources, we aimed to confirm the accuracy and reliability of our dataset. Leveraging information from open sources enhances the credibility of our findings and strengthens the validity of our analyses.

Utilizing multi-objective optimization is widely discussed in the scientific literature, as exemplified by Mannodi et al. [141], who developed two Monte Carlo algorithms to pinpoint the Pareto front in the chemical space of dielectric polymers, optimizing both the bandgap and dielectric constant. Using machine learning on a dataset from density functional theory calculations, they created surrogate models for four-block polymers and extended their applicability.

Garcia et al. [142] developed an ANN to predict thermal and electrical conductivity in HDPE–carbon particle composites. ANNs served as objective functions in a multi-objective GA to optimize composite design parameters. The GA generates Pareto-optimal solutions [143] for maximizing thermal conductivity and minimizing electrical conductivity. This approach offers a systematic framework for optimizing polymer composite properties efficiently.

The approach taken in this study showcases several novel aspects, particularly in the optimization of the predictive accuracy and inference time for the physical properties of TPCMs by analyzing the components they are produced from, like the properties of the yarns in the warp and weft directions, as well as the fabrics made from these yarns by utilizing MOPSO, SPEA2, and NSGA-II to achieve a more efficient exploration of the high-dimensional parameter space. This resulted in more accurate predictions and reduced inference times, which is a significant improvement over traditional optimization methods by reducing the need for time-consuming and expensive physical experiments. By accurately predicting the properties of new composite materials based on existing data, we can streamline the development process of new TPCM products.

This method also offers the potential for incorporating multiphysical computational methods, which can further enhance the accuracy and relevance of the predictions. This integration supports the development of comprehensive models that account for various physical phenomena simultaneously.

Despite the promising results, several challenges and limitations must be acknowledged. One notable limitation is the availability and quality of data. While efforts were made to collect comprehensive datasets, variations in the data sources and measurement techniques may introduce inconsistencies and biases.

A trade-off exists between model interpretability and predictive performance. While complex ANNs may achieve superior predictive accuracy, they often lack interpretability, hindering insights into the underlying physical mechanisms. Future research should explore techniques for enhancing model interpretability without compromising performance, such as layerwise relevance propagation [144] and feature attribution methods.

The generalization ability of predictive models is crucial for their practical applicability across diverse TPCM systems and processing conditions. Robust validation strategies, including cross-validation and out-of-sample testing [145], are essential for assessing model generalization and ensuring reliable predictions in real-world scenarios.

Various frameworks exist for multi-physics modeling. For instance, PERMIX [146] is an open-source framework designed for multiscale modeling and simulation of fractures in solids, utilizing the extended finite-element method (XFEM) and integrating with libraries like LAMMPS and ABAQUS. It accommodates both semi-concurrent and concurrent multiscale methods for detailed fracture simulations.

Alternatively, Liu et al. [147] introduced a hybrid ML approach employing an ANN and PSO to predict the thermal conductivity of polymeric nanocomposites (PNCs). By combining the ANN for modeling and PSO for optimization, they achieved superior predictive performance compared to traditional ANNs. Key input parameters included the thermal conductivity of fibers and matrix, Kapitza resistance, volume fraction, and the aspect ratio, with the output being the composite’s macroscopic thermal conductivity.

Moreover, N. Vu-Bac [148] integrated molecular dynamics (MD) simulations to examine the impact of the single-walled carbon nanotube (SWCNT) radius, temperature, and pulling velocity on the interfacial shear stress (ISS) of PNCs by assessing the influence of uncertain input parameters on ISS prediction by computing partial derivatives via averaged local sensitivity analysis (SA) and employing surrogate models (polynomial regression, moving least squares, and hybrid models) for computational efficiency.

Ilyani Abu et al. [149] utilized unit cells and evolutionary algorithms to forecast the geometric characteristics and elastic properties of woven fabric composites, through optimizing elastic properties within these unit cells to accurately predict mechanical behavior. TexGen generated the weave patterns; ABAQUS was used to conduct the simulations; finite-element (FE) analysis estimated the effective elastic properties of the yarn. The parameter studies delved into the effects of various geometric parameters, facilitating the selection of an optimal parameter set for composite performance.

The Parametric Deep Energy Method (P-DEM) for elasticity problems incorporating strain gradient effects was suggested in [150]. Utilizing physics-informed neural networks [151] (PINNs), the authors optimized a cost function associated with potential energy, eliminating the need for classical discretization. By defining a parametric/reference space akin to isoparametric finite elements, and leveraging NURBS [152] basis functions, P-DEM achieves efficient computation of the total potential energy.

Accurate predictive models for TPCM properties offer significant implications for materials’ design, process optimization, and product performance prediction. By leveraging these models, engineers and designers can expedite material development cycles, optimize manufacturing processes, and tailor materials’ properties to meet specific application requirements.

Future research directions may include the incorporation of advanced feature engineering techniques, such as image-based analysis and spectral imaging, to extract rich structural and compositional information from TPCM samples. Additionally, the integration of physics-based models with machine learning approaches could enhance predictive accuracy and facilitate mechanistic understanding of material behavior.

## 5. Conclusions

In conclusion, our study highlights the significant potential of ML and optimization techniques in advancing the predictive modeling of TPCM properties. By leveraging SVMs and ANNs as powerful modeling tools, we successfully optimized their hyperparameters using state-of-the-art optimization algorithms, including MOPSO, NSGA II, and SPEA 2. Through this approach, we have demonstrated the effectiveness of integrating advanced ML techniques with optimization methodologies to enhance the accuracy, robustness, and applicability of predictive models for TPCM properties. By addressing key challenges and exploring innovative methodologies, researchers can further propel advancements in material science and engineering, ultimately facilitating the development of high-performance textile polymer composites for diverse applications.

## Figures and Tables

**Table 1:** Comparison of optimized values for the SVM and ANN using MOPSO, SPEA2, and NSGA-II.

ML Model	Optimized Value	MOPSO	SPEA2	NSGA-II
SVM	accuracy	0.878	0.876	0.899
	inference time, ms	0.88	0.85	0.78
	Parameters (C, γ)	(1.0, 0.1)	(1.2, 0.08)	(0.9, 0.15)
ANN	accuracy	0.902	0.901	0.898
	inference time, ms	0.42	0.36	0.43
	Architecture (layers, neurons, activation)	(4, [4, 28, 20, 12], ReLU)	(4, [2, 16, 8, 16], sigmoid)	(3, [6, 4, 8], tanh)

## Data Availability

All the data used in this study are available in the TPCM repository. The repository contains a comprehensive dataset comprising the properties of the TPCMs, meticulously compiled from experimental data. Researchers interested in accessing the data can find them in the provided repository.
